# Is There Sufficient Evidence for Criticality in Cortical Systems?

**DOI:** 10.1523/ENEURO.0551-20.2021

**Published:** 2021-04-15

**Authors:** Alain Destexhe, Jonathan D. Touboul

**Affiliations:** 1 Paris-Saclay Institute of Neuroscience, Centre National de la Recherche Scientifique, 91198 Gif-sur-Yvette, France; 2 Department of Mathematics and Volen Center for Complex Systems, Brandeis University, Waltham, MA 02453

**Keywords:** cerebral cortex, dynamics, spontaneous activity

## Abstract

Numerous studies have proposed that specific brain activity statistics provide evidence that the brain operates at a critical point, which could have implications for the brain’s information processing capabilities. A recent paper reported that identical scalings and criticality signatures arise in a variety of different neural systems (neural cultures, cortical slices, anesthetized or awake brains, across both reptiles and mammals). The diversity of these states calls into question the claimed role of criticality in information processing. We analyze the methodology used to assess criticality and replicate this analysis for spike trains of two non-critical systems. These two non-critical systems pass all the tests used to assess criticality in the aforementioned recent paper. This analysis provides a crucial control (which is absent from the original study) and suggests that the methodology used may not be sufficient to establish that a system operates at criticality. Hence whether the brain operates at criticality or not remains an open question and it is of evident interest to develop more robust methods to address these questions.

Whether the brain operates at criticality or not has been debated since Beggs and Plenz (2003) reported that power-law statistics of firing patterns show similar behavior to that of physical systems at a phase transition. This suggestion sparked the interest of theoreticians, in search of a rigorous validation of this hypothesis and for a theory of the origin and implications of criticality in neural systems (Mora and Bialek, 2011). In tandem, experimentalists searched for evidence of criticality in neural systems and physiological or pathologic brain states (Hahn et al., 2010; Friedman et al., 2012; Massobrio et al., 2015). Some papers also hypothesized that criticality was a hallmark of healthy brain function and optimal information processing (Beggs, 2008; Shew et al., 2009; Shew and Plenz, 2013).

However, many theoretical studies have shown that the evidence provided for criticality in experiments is not specific to critical systems. Some papers proposed that simple phenomena could play a role in the emergence of the positive detections of criticality reported in the literature. For example, artifacts of thresholding noisy signals (Touboul and Destexhe, 2010), intermittent activity (Miller, 1957), and how that may affect high-dimensional neural data (Laurence et al., 2014) or large-scale interacting networks (Schwab et al., 2014; Touboul and Destexhe, 2017) have been shown to generate purported signatures of critical behavior. One of the key difficulties related to the criticality hypothesis is the lack of a univocal statistical test, which in turn points to the difficulty in identifying a specific type of phase transition associated with the putative critical dynamics.

With the aim of bringing together theoretical findings and experimental data, we investigate the conclusions of a recent paper reporting remarkable power-law scalings on an extensive dataset. The paper in question analyzes neural data recorded in various species, with distinct preparations and different brain states using a unified methodology (Fontenele et al., 2019). Strikingly, data ranging from freely moving or anesthetized mammals to *ex vivo* preparations of reptile nervous system or cultured slices of rat cortex all show common scaling in a specific activity regime. The authors interpret this common scaling as an unspecified critical regime (which is said to be distinct from the classical mean-field directed percolation model). The extraordinary consistency of the scalings observed in different brain states and preparations is very surprising. In particular, the fact that in vitro neuronal cultures and deeply anesthetized states are found to be critical raises some intriguing questions about how that “critical” state relates to optimal information processing in the brain.

It is thus essential to determine whether the evidence provided in existing experimental studies is sufficient to conclude that the system studied operates at criticality. To assess criticality, Fontenele et al. (2019) used a test based on the relationship between power-law scaling exponents of neuronal avalanches in experiments and in a model at criticality inspired from classical crackling-noise systems. However, the authors in Fontenele et al. (2019) did not provide any control (non-critical systems) to assess whether the methodology distinguishes critical and non-critical models of neural networks. In fact, all the systems they considered passed their criticality tests.

The methodology used by Fontenele et al. (2019) consists of fitting the distribution of neuronal avalanche size (exponent *τ*) and duration (exponent *τ_t_*) with power-laws truncated to a cutoff. The fit is validated by comparing the Akaike information criterion (AIC) associated with the AIC of a log-normal fit. The authors found acceptable support for power-law distributions (compared with the log-normal distribution), but noted that exponents found are not compatible with the mean-field directed percolation systems generally used as a reference to assess criticality. Nonetheless, they classified systems as critical when exponents satisfy Sethna’s crackling relationship:

(1) $$\frac{\tau_{t}-1}{\tau -1}=a,$$

where *a* is the power-law scaling of the average avalanche size as a function of duration. Renormalization theory shows that this scaling is universal, at criticality, for a specific class of systems called crackling noise systems. The choice of this test implicitly assumes that the neuronal systems belong to the universality class of crackling systems, which to date remains an open question and has not been established. In fact, the authors refer to our own theoretical paper (Touboul and Destexhe, 2017) as support for the use of this relationship to distinguish critical from non-critical systems. However, the results in our paper do not support the test performed in Fontenele et al. (2019). We showed that for two non-critical models all hallmarks used to identify criticality in the experimental literature were satisfied. These counterexamples would not satisfy Sethna’s relationship (1) in the thermodynamic limit and for the scaling of the tails of the distributions of avalanches. We do not make any claim about the scaling related to the bulk of avalanche distributions, and our results should not be construed as demonstrating that systems not satisfying (1) are critical [or that those for which (1) is not satisfied are not critical]. To assess whether the two non-critical systems studied in Touboul and Destexhe (2017) indeed provide signatures distinct from the neural systems analyzed in Fontenele et al. (2019), we replicated the analysis in Fontenele et al. for the two models studied in Touboul and Destexhe [2017; the Brunel network; Brunel (2000) and a stochastic surrogate (Touboul and Destexhe (2017)]. We investigated how fitting the bulk up to a cutoff affects the tests and performed extensive simulations, computed avalanche distributions for size and duration, fitted power-law distributions with various cutoffs, and used the AIC difference test proposed in Fontenele et al. (2019) to validate the power-law fits (100% of the *n *=* *32,000 distributions considered in Fig. 1 passed the test). We next checked, based on the fitted exponents and for each set of threshold, whether (1) was statistically valid using a two-sample *t* test on multiple independent repetitions of the simulation. We found a connected region of pairs of threshold values for which the statistics are statistically significantly consistent with (1), and would therefore be classified as critical by the criterion used in Fontenele et al. (2019).

**Figure 1.**
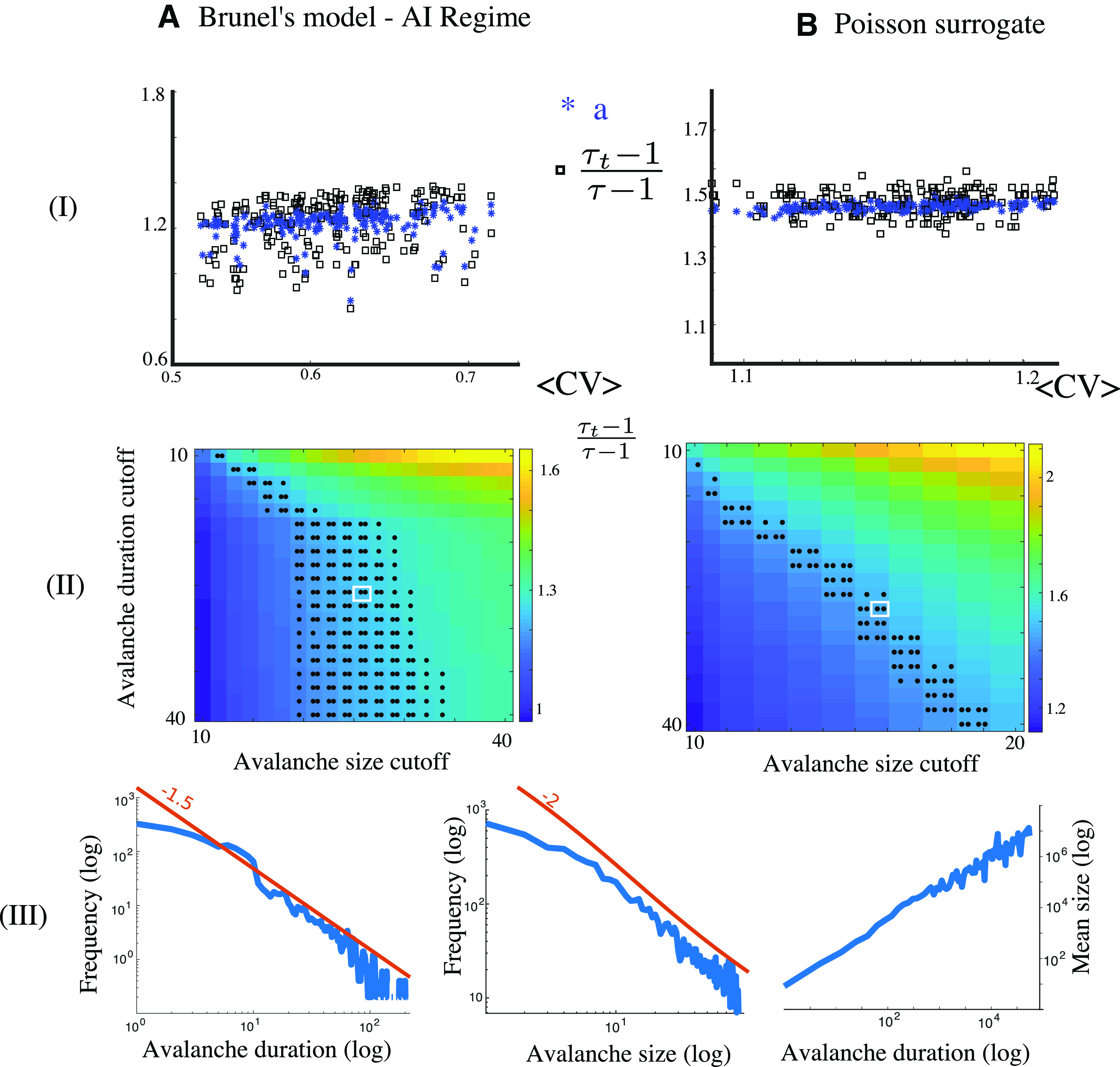
A total of 200 simulations of the Brunel model (***A***, left, parameters as in Touboul and Destexhe, 2017, Fig. 7) and Poisson surrogate (***B***, right, Ornstein–Uhlenbeck rate with randomly chosen coefficients). Top row (***I***), Two examples of networks classified as critical by the criteria in Fontenele et al. (2019). Middle (***II***), A multitude of combinations of cutoffs yield results compatible with Sethna’s relationship (two-sample *t* test, MATLAB function ttest2, comparing the distribution of ratios and a, *p < 0.01, **p < 0.05, for *n* = 14 instances as in Fontenele et al., 2019). Bottom (***III***), Example of distributions of avalanche durations (left), size (middle), or average size versus duration in logarithmic scale, used to obtain the statistics in ***I***, ***II***, compare to Fontenele et al. (2019; their Figs. 1*F*,*G*, 2*C*).

These counterexamples to the test used in Fontenele highlight the fact that the evidence provided is not sufficient to establish that the data they analyzed is from a system at criticality. The truncation of the data performed in Fontenele et al. (2019; with thresholds as low as 15–25 duration bins) is a good practice and inevitable for experimental datasets. However, truncation of the data may substantially alter the statistics, particularly when it comes to estimating the tails of a distribution. In Fontenele et al. (2019), the truncation is a crucial step in the methodology as fits are performed from the smallest observable avalanche, and therefore small cutoffs will significantly impact up to the cutoff distribution of small avalanches and are less likely to accurately catch the behavior of the tails. Therefore, while the authors do report evidence that the brain, in some regimes, shows statistics that are consistent with a given type of critical system, they did not establish that the brain operates at criticality, because the methodology used appears insufficient to distinguish critical from non-critical systems.

Brunel’s model is a well-known network model displaying activity states relevant to cortical activity. The fact that this model can satisfy all aspects of the analysis in Fontenele et al. (2019) away from criticality suggests that the most parsimonious explanation for the data does not require criticality, a regime that entails fine tuning of physiology parameters or homeostatic mechanisms for constraining self-organization. Moreover, such basic phenomena appear consistent with the ubiquity of these observations in a variety of neural systems from awake animals to reptile *ex vivo* neurons. This report underlines once more that the criticality hypothesis is yet to be established, and that rigorous methods should be developed. To make progress in this area, experimentalists and theoreticians should come together to make precise definitions of the type of criticality that could arise in the brain and establish rigorous, univocal tests for that criticality.

## Code Availability

The program code used to reproduce the figure of the paper is available in Destexhe and Touboul (2021).

## Synthesis

Reviewing Editor: William Stacey, University of Michigan Decisions are customarily a result of the Reviewing Editor and the peer reviewers coming together and discussing their recommendations until a consensus is reached. When revisions are invited, a fact-based synthesis statement explaining their decision and outlining what is needed to prepare a revision will be listed below. The following reviewer(s) agreed to reveal their identity: Mark Kramer, Viktor Jirsa.

The reviewers agreed that this short article has provided a valid counterexample, in which a non-critical system passes all the tests for criticality. These arguments show the insufficiency of a test of criticality of brain dynamics. However, there are several concerns that should be addressed prior to being acceptable for publication.

1. Authors need to provide code for replication. Please provide in a public repository (e.g., github) all of the code needed to simulate the models and produce the results of this paper.

2. There are many spelling and grammatical errors throughout, which make it difficult to follow the (important) points of this paper.

A few specifics:

”...are evidence sufficient to claim that the regime associated is associated with a critical regime from a realistic model.. “ please rephrase to be more clear, avoid double use of associated.

”...where a the scaling of the average avalanche size as a function of duration.” Add “a is the scale...”

"In fact, the authors refer to our own theoretical paper [5] as a support for the use of this relationship to distinguish critical from non-critical system” Wrong citation, we think the reference should be [14]?

"We therefore thoroughly replicated the analysis in Fontenele in the two models studied

in [15] (the Brunel network [4] and a stochastic surrogate [15]) and investigated fitting the bulk up to a cut affects the tests. We therefore performed extensive simulations, ...” Rephrase, avoid use of two time “We therefore..”

”...yet this can affect substantially the statistics of the tails and when fits are done the smallest observable avalanche. “ Rephrase please

3. Some further description of the simulated results is necessary. Specifically:

- “and used the AIC difference test proposed in [5] to validate the power-law fits (100% of the n = 32 000 distributions considered in Fig. 1 passed the test).” Could this be show in a Figure, perhaps replicating something like Fig 1H of [Fontenele et al 2019]?

- “We next checked, based on the fitted exponents, whether (1) was statistically valid using a two-sample t-test.” Please provide all details of what is being tested, exactly. Is there a correction for multiple comparisons? Perhaps show a histogram of the p-values (without correction).

- “We found that a multitude of those counter-examples are consistent with (1), and would therefore be classified as critical by the criterion used in [5].” Please quantify this result. What percentage is “a multitude”?

- In general, it's difficult to understand Figure 1. One strategy could be to mimic more elements of Figures 1 and 2 in [Fontenele et al 2019]. If the authors clearly show their simulations produce features that look very similar to the Figures in [Fontenele et al 2019], that would help the emphasize the results.

- The authors state that “One of the key difficulty to date related to the criticality hypothesis is the lack of a univocal statistical test,”. In the conclusions, could the authors speculate on the essential features of such a test?

4. In [Fontenele et al 2019] there’s an emphasis on the intersection of the left and right sides of the Sethna’s crackling relationship equation, eg., Figures 2A,B,E,F of [Fontenele et al 2019]. In this eNeuro submission, the authors plot two examples of this relationship in Figure 1, top. There’s no clear intersection point; while the plots in [Fontenele et al 2019] look like an “X", the plots here in Figure 1 look like two overlapping lines. Is this difference related to the criticality of a system? In other words, do we see an “X” in [Fontenele et al 2019] because those systems are critical, and do we fail to see this “X” here because the simulated systems are not critical? Would this difference in the shapes of the plots support the conclusions in [Fontenele et al 2019] - only a critical system would produce an “X” in these plots? (This question is from a novice in critical systems.)

5. First sentence in abstract is unfortunately wrong and needs to be corrected. Operation close to criticality is not identical to self-organized criticality, the latter requiring a self-tuning process assuring attraction to the critical point.
